# Multimodal EEG–HRV assessment of individual regulatory patterns: a perturbation-based approach toward precision psychiatry

**DOI:** 10.3389/fpsyt.2026.1851256

**Published:** 2026-08-24

**Authors:** Zsoltné Hauber, Sándor György Fekete, Elek Dinya, István Karsai, Gábor Elbert

**Affiliations:** 1 Doctoral School of Health Sciences, Faculty of Health Sciences, University of Pécs, Pécs, Hungary; 2 Department of Laboratory Animal Science and Animal Protection, University of Veterinary Medicine, Budapest, Hungary; 3 Institute of Digital Health Sciences, Faculty of Public Services, Semmelweis University, Budapest, Hungary; 4 Sports and Physical Education Center, Medical School, University of Pécs, Pécs, Hungary; 5 Institute of Physiotherapy and Sport Science, Faculty of Health Sciences, University of Pécs, Pécs, Hungary

**Keywords:** cortico–autonomic coupling, cortico–autonomic response patterns, EEG–HRV integration, individual variability, neurophysiological Biomarkers, precision psychiatry, wearable EEG

## Abstract

**Background:**

Contemporary psychiatric research increasingly emphasizes the need for objective, biologically grounded markers to complement symptom-based diagnostic frameworks. While electroencephalography (EEG) has been widely used to investigate neural dynamics associated with attention, arousal, and cognitive control, these approaches are typically brain-centric and rely on static or resting-state paradigms. Integration of autonomic dynamics remains limited despite theoretical models highlighting brain–body interactions.

**Methods:**

In the present pilot study, we employed a multimodal, perturbation-based paradigm combining wearable EEG and heart rate variability (HRV) to examine cortico–autonomic regulatory dynamics. Ten healthy participants were exposed to brief auditory conditions (silence, noise-based stimulation, and binaural-beat auditory input). Frontal upper-beta activity (β_2_; 21–30 Hz) and vagally mediated HRV indices (RMSSD, HF power) were analyzed to characterize within-subject regulatory responses across conditions.

**Results:**

Condition-related variation was observed descriptively in both cortical and autonomic measures, although no statistically significant group-level effects were detected. Substantial inter-individual variability emerged in the direction and magnitude of these responses. Notably, substantial inter-individual variability emerged in both the direction and magnitude of physiological responses. In several participants, coordinated changes between EEG and HRV measures suggested multimodal cortico–autonomic response patterns, whereas others exhibited divergent or minimal response profiles. Coordinated decreases in frontal β_2_ activity together with increases in RMSSD were observed in 4 of the 10 participants. Given the exploratory nature of the study and the limited sample size, these observations should be interpreted as preliminary and hypothesis-generating.

**Conclusion:**

These findings support the potential utility of multimodal, stimulus-dependent approaches for characterizing dynamic brain–body responses beyond static biomarkers. The observed multimodal response configurations are consistent with coordinated cortico–autonomic response patterns and provide a useful framework for generating hypotheses regarding individual variability in brain–body regulation. As an exploratory pilot study, the present findings require validation in larger and clinically diverse cohorts before conclusions regarding physiological subgroups or clinical applications can be drawn. This perturbation-based multimodal EEG–HRV framework may contribute to future physiology-informed precision psychiatry research by providing a methodological foundation for characterizing individual brain–body response variability.

## 1 Introduction

Contemporary psychiatric research is increasingly moving toward biologically informed approaches that complement traditional symptom-based diagnostic frameworks by incorporating objective measures of brain and physiological function (1, 2). Rather than assuming that diagnostic categories reflect homogeneous biological mechanisms, emerging precision psychiatry frameworks emphasize the importance of understanding individual differences in neurophysiological regulation that may contribute to clinical heterogeneity (2). Recent work in affective and biological psychiatry further suggests that variability in physiological and interoceptive processes may represent an important dimension of psychiatric heterogeneity rather than simply reflecting measurement noise (3, 4). Within this context, multimodal physiological assessment has gained increasing attention because coordinated brain–body processes cannot be adequately characterized using symptom ratings or isolated biological measures alone. Consequently, there is growing interest in methodological approaches capable of examining dynamic interactions between neural and autonomic systems as potential sources of individualized physiological variability.

Electroencephalography (EEG) and heart rate variability (HRV) are among the most widely used non-invasive techniques for investigating cortical and autonomic processes involved in cognitive, emotional, and regulatory functioning (5–8). EEG provides high temporal resolution for assessing cortical dynamics and has long been used to investigate attentional processing, stimulus evaluation, and large-scale neural network activity (8–10). In parallel, vagally mediated HRV indices provide validated measures of autonomic flexibility and adaptive physiological regulation (6, 7). Although both modalities have substantially advanced the understanding of human neurophysiology, they are still most frequently investigated independently or within static experimental paradigms. Consequently, relatively little is known about how cortical and autonomic responses interact dynamically within individuals during standardized experimental perturbations, despite theoretical models emphasizing integrated brain–body regulation (5, 11).

Affective regulation is increasingly conceptualized as a dynamic and context-dependent process arising from interactions between prefrontal cortical systems, attentional networks, and autonomic regulatory mechanisms (12–14). Within this framework, physiological measures such as frontal EEG activity and HRV provide complementary information regarding coordinated brain–body regulation rather than isolated neural or autonomic function (5, 7). The Neurovisceral Integration Model proposes that adaptive regulation emerges through reciprocal interactions between cortical control networks and vagally mediated autonomic mechanisms, supporting flexible behavioral, cognitive, and emotional adaptation (5, 11). However, relatively few studies have examined these interactions using synchronized multimodal measurements obtained during standardized perturbation paradigms specifically designed to characterize individual physiological response variability rather than average group effects.

Most contemporary neurophysiological studies remain predominantly state-oriented, focusing on resting-state recordings or isolated responses to experimental stimuli. Although these approaches provide valuable information regarding momentary neural and autonomic activity, they may not fully capture how cortical and autonomic systems dynamically coordinate during adaptation to controlled environmental challenges (3, 14). Likewise, growing evidence suggests that identical sensory inputs may elicit substantially different physiological responses across individuals, reflecting variability in regulatory organization rather than uniform stimulus-driven effects (4, 15, 16). Characterizing such inter-individual response variability may therefore complement conventional group-level analyses and contribute to a more comprehensive understanding of coordinated brain–body regulation.

Parallel developments in network neuroscience have emphasized the role of salience and attentional control systems in coordinating adaptive responses to changing environmental demands (17–19). Frontal beta-band activity, particularly within the upper-beta frequency range, has been associated with attentional engagement, cognitive effort, and adaptive regulation of cortical processing (20, 21). At the autonomic level, vagally mediated HRV indices, including RMSSD and high-frequency (HF) power, represent well-established markers of parasympathetic regulation and physiological flexibility (6, 7). Simultaneous assessment of these complementary physiological domains therefore provides an opportunity to examine coordinated cortico–autonomic responses using an integrated multimodal framework.

Recent advances in wearable neurophysiology have further expanded the feasibility of synchronized multimodal EEG and HRV assessment outside traditional laboratory environments (22, 23). Within this context, brief standardized auditory perturbations provide a practical experimental model because they enable controlled modulation of sensory processing while minimizing task-related cognitive demands (24, 25). Rather than being viewed primarily as therapeutic interventions, such perturbations can be employed as standardized experimental probes for examining coordinated cortico–autonomic responses under identical experimental conditions. This perturbation-based approach is intended to characterize individual physiological response variability and generate hypotheses regarding multimodal brain–body regulation rather than to establish predefined physiological classifications.

Building upon this conceptual framework, the present exploratory pilot study employed a perturbation-based multimodal approach integrating synchronized wearable electroencephalography (EEG) and heart rate variability (HRV) recordings during standardized auditory conditions. Rather than considering auditory stimulation primarily as an intervention, the experimental paradigm utilized controlled sensory perturbations as standardized physiological probes for examining coordinated cortico–autonomic responses under identical experimental conditions. This approach was designed to characterize participant-level physiological response variability beyond conventional group-level analyses while remaining consistent with contemporary concepts of integrated brain–body regulation (5, 11).

Recent developments in precision psychiatry have emphasized the importance of biologically informed frameworks capable of complementing symptom-based diagnostic approaches while acknowledging substantial inter-individual variability (2). Likewise, recent work on biologically informed psychiatric subgroups has highlighted the need for objective physiological characterization while emphasizing that such approaches require rigorous validation in large and clinically diverse populations before clinically meaningful classifications can be established (26, 27). In keeping with these perspectives, the present study does not seek to identify physiological subgroups or establish stable regulatory phenotypes. Instead, it evaluates whether synchronized multimodal EEG–HRV recordings can provide an exploratory framework for describing coordinated cortico–autonomic response variability during standardized sensory perturbations.

Accordingly, the present study was designed to examine whether synchronized multimodal EEG and HRV recordings obtained during standardized auditory perturbations could characterize coordinated physiological responses at the individual level. Particular emphasis was placed on describing multimodal response variability, examining convergence between cortical and autonomic measures, and evaluating the methodological feasibility of this perturbation-based framework. Because of the exploratory nature of the study and the limited sample size, all observed response configurations were interpreted as candidate physiological response patterns intended to generate hypotheses for future investigation rather than as empirically established physiological phenotypes or clinically applicable classifications.

The following exploratory research questions guided the analysis:

RQ1. What degree of inter-individual variability is observed in cortico–autonomic responses to standardized auditory perturbations?
RQ2. To what extent do directional changes in frontal β_2_ activity correspond with changes in vagally mediated HRV indices at the participant level?
RQ3. Do structured and non-structured auditory conditions show descriptively different multimodal response tendencies across participants?

A secondary methodological objective was to evaluate whether a perturbation-based multimodal EEG–HRV framework could provide a feasible approach for characterizing coordinated brain–body response variability and generating hypotheses relevant to future physiology-informed precision psychiatry research.

**Figure 1:**
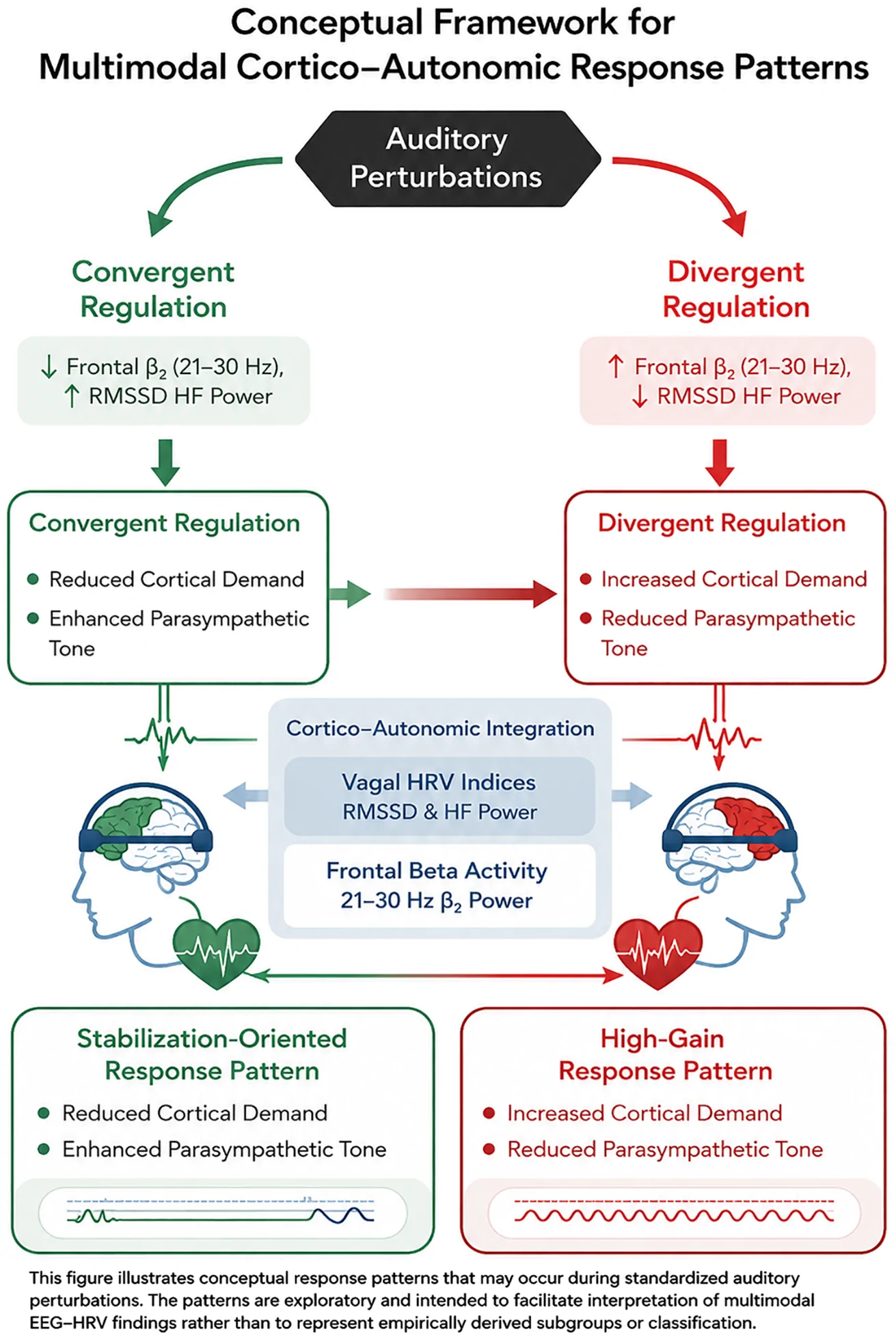
Conceptual framework illustrating hypothetical multimodal cortico–autonomic response patterns during standardized auditory perturbations. The diagram presents two conceptual regulatory configurations characterized by convergent or divergent changes in frontal β_2_ activity (21–30 Hz) and vagally mediated heart rate variability (RMSSD and HF power). These response patterns are intended solely to facilitate interpretation of multimodal EEG–HRV findings and should not be interpreted as empirically derived physiological subgroups, validated phenotypes, or diagnostic classifications. The framework is proposed as a conceptual model to support future hypothesis generation and precision-oriented psychophysiological research.

**Figure 1** summarizes the conceptual framework underlying the present exploratory pilot study. The illustrated response configurations are conceptual and intended solely to facilitate interpretation of multimodal findings. They should not be interpreted as empirically derived physiological subgroups or statistically validated phenotype classifications.

Brief auditory contexts, including structured and non-structured stimuli, engage interacting cortical and autonomic regulatory systems. Frontal upper-beta activity (β_2_; 21–30 Hz) is depicted as an index of cortical arousal dynamics, while heart rate variability (HRV) measures such as RMSSD and high-frequency (HF) power reflect vagally mediated autonomic regulation.

The model schematically illustrates how different patterns of coordinated EEG–HRV responses may be interpreted within the proposed conceptual framework. Convergent patterns, characterized by reduced frontal β_2_ activity alongside increased vagal indices, are interpreted as stabilization-oriented regulatory tendencies associated with reduced cortical demand and enhanced parasympathetic engagement. In contrast, divergent patterns, involving increased β_2_ activity and reduced vagal indices, reflect regulatory configurations associated with elevated cortical demand and reduced parasympathetic tone.

Rather than implying discrete categories, the model represents a conceptual framework for understanding variability in cortico–autonomic coupling as a continuum of regulatory tendencies. This multimodal perspective provides a conceptual framework for characterizing individual differences in coordinated brain–body responses relevant to neurophysiological and psychiatric variability.

The present framework conceptualizes standardized auditory perturbations as experimental probes for examining coordinated cortico–autonomic responses under controlled conditions. Rather than identifying stable physiological subgroups, the approach is intended to facilitate exploratory characterization of multimodal response variability and generate hypotheses for future validation in larger and clinically diverse cohorts.

Guided by this conceptual framework and the Neurovisceral Integration Model (5, 11), the present exploratory pilot study employed synchronized wearable EEG and HRV measurements during standardized auditory perturbations to characterize participant-level cortico–autonomic responses. Rather than establishing physiological classifications, the study focused on describing multimodal response variability and generating hypotheses for future physiology-informed psychiatric research.

## 2 Methods

### 2.1 Participants

This exploratory pilot study included ten healthy adult volunteers (4 males and 6 females; age range: 26–66 years; mean age: 42.3 ± 13.8 years), who were recruited on a voluntary basis. Inclusion criteria comprised normal hearing ability, absence of neurological, psychiatric, or cardiovascular diagnoses, and no use of medication affecting central or autonomic nervous system functioning. Participants were right-handed according to standardized self-report criteria.

All participants provided written informed consent in accordance with the Declaration of Helsinki. Ethical approval was granted under protocol BM/9282-3/2023 (Hungary).

### 2.2 Experimental design: sensory perturbation paradigm

A within-subject perturbation design was employed to examine cortico–autonomic regulatory dynamics. Rather than evaluating stimulus-specific emotional responses, the experimental structure was designed to expose intrinsic individual regulatory responses through controlled sensory perturbations.

Each participant completed three brief eyes-closed conditions:

1. Silence (baseline regulatory state).
2. Non-structured atonal noise context (high-entropy perturbation).
3. Structured 16-Hz binaural-beat auditory context (rhythmic entrainment perturbation).

Each condition lasted three minutes and was divided into three consecutive epochs (early, middle, late) to assess within-session stability.

The paradigm conceptualizes auditory contexts as regulatory perturbations capable of eliciting system-specific responses within cortico–autonomic regulatory networks. Analytical emphasis therefore focused on within-person response directionality and multimodal convergence rather than group-average effects.

### 2.3 Auditory perturbation contexts

Auditory contexts were selected to represent distinct regulatory perturbation profiles differing in spectral structure and predictability:

- Silence: eyes-closed quiet baseline reflecting intrinsic regulatory tone.
- Noise context: atonal, spectrally irregular acoustic texture representing high-entropy sensory input.
- 16-Hz binaural-beat context: tonal melody embedding a dichotic 16-Hz beat (440 Hz left channel; 456 Hz right channel), representing structured rhythmic entrainment.

All stimuli were presented as uncompressed WAV files via high-fidelity over-ear headphones at 60–65 dB SPL in an acoustically controlled environment (<35 dB ambient noise).

### 2.4 Wearable EEG acquisition

Electroencephalographic activity was recorded using a Muse-2 wearable EEG system (23) with a sampling rate of 256 Hz, 12-bit resolution, channels AF7, AF8, TP9, and TP10, and FPz reference.

Data segments were retained only when signal quality index (SQI) exceeded 90%. The minimal frontal montage was selected to capture regulatory arousal dynamics associated with prefrontal networks while preserving ecological feasibility.

Frontal upper-beta activity (β_2_; 21–30 Hz) was used as an operational marker of cortical arousal dynamics.

### 2.5 HRV acquisition

Cardiac autonomic activity was recorded using a Polar RS800 system with 1-ms R–R interval resolution. HRV analysis was conducted using Kubios HRV Premium with adaptive artifact correction.

Primary autonomic indices included:

- RMSSD (parasympathetic activity).
- HF power (0.15–0.40 Hz).
- LF/HF ratio (reported as an exploratory composite HRV index).

Primary interpretation focused on RMSSD and HF power as indices related to vagally mediated cardiac regulation. LF/HF was treated as a secondary exploratory measure and was not interpreted as a direct measure of sympathovagal balance.

RMSSD and HF power were interpreted as indices of vagally mediated cardiac regulation, whereas LF/HF was retained as a secondary exploratory measure.

### 2.6 EEG processing and feature extraction

EEG preprocessing included band-pass filtering (1–40 Hz) and 50-Hz notch filtering. Artifact-contaminated segments were excluded based on SQI thresholds and visual inspection.

Frontal β_2_ power was estimated using Welch spectral analysis (4-s windows, 50% overlap). A frontal asymmetry index was calculated:

AIβ_2_ = ln(β_2__AF8) − ln(β_2__AF7).

Positive values indicate relative right-frontal dominance. Baseline-corrected change scores were computed relative to the silence condition.

Multimodal response patterns were examined by integrating cortical and autonomic changes at the individual level, with emphasis on directionality and within-person consistency across conditions.

Frontal β_2_ asymmetry between the homologous frontal recording sites AF7 and AF8 was quantified using a log-transformed power ratio:

AIβ_2_ = ln(PowerAF8,β_2_) − ln(PowerAF7,β_2_).

Positive values indicate relatively greater β_2_ power at AF8 than at AF7. Baseline-corrected change scores were calculated as ΔAIβ_2_ = AIβ_2_(condition) − AIβ_2_(silence).

This site-level asymmetry measure describes relative differences between the recorded frontal electrodes and should not be interpreted as evidence of cortical source localization or whole-hemisphere functional dominance.

Frontal β_2_ asymmetry was quantified using a log-transformed asymmetry index, AIβ_2_ = ln(AF8β_2_) − ln(AF7β_2_). Condition-specific changes were calculated relative to silence.

The present multimodal analysis used a log-transformed asymmetry index; therefore, the numerical ΔAIβ_2_ values should not be directly compared with values obtained using normalized difference indices, although both measures preserve the direction of relative AF8–AF7 modulation.

### 2.7 Statistical strategy

Statistical analyses were performed using IBM SPSS v29.

Analytical emphasis prioritized:

- within-person response directionality.
- cross-context stability.
- multimodal convergence patterns.

Procedures included:

- Shapiro–Wilk normality testing.
- Friedman repeated-measures tests.
- Wilcoxon *post hoc* comparisons.
- effect size estimation (Cohen’s dz).

The primary endpoint was baseline-corrected frontal asymmetry (ΔAIβ_2_). Response profiles were qualitatively described based on observed directionality patterns between EEG and HRV changes.

This study should therefore be considered an exploratory pilot investigation aimed at identifying candidate regulatory architectures rather than establishing population-level effects.

The conceptual framework underlying the present analysis was previously documented in a time-stamped repository record (Zenodo), establishing methodological provenance prior to journal submission.

To examine cortico–autonomic regulatory dynamics, multimodal convergence between EEG and HRV changes was assessed at the individual level.

Because of the exploratory pilot design and the limited sample size, the analytical strategy focused primarily on within-subject response characterization and descriptive evaluation of multimodal EEG–HRV convergence rather than formal identification of physiological subgroups. No clustering analyses or phenotype classification procedures were performed. Accordingly, the multimodal response categories presented in this study should be interpreted as conceptual descriptors intended to facilitate physiological interpretation and generate hypotheses for future investigation rather than as empirically validated biological classifications (28, 29).

Accordingly, the analytical strategy was designed to characterize multimodal response variability and generate hypotheses for future validation rather than to establish population-level physiological classifications.

## 3 Results

Across experimental conditions, stimulus-dependent modulation was observed in both EEG-derived frontal activity and HRV indices, indicating engagement of cortical and autonomic regulatory processes.

Notably, substantial inter-individual variability was evident in both the direction and magnitude of responses, suggesting heterogeneous regulatory response tendencies across participants.

In several cases, coordinated shifts between cortical and autonomic measures were observed, indicating potential coupling between neural and physiological regulatory systems.

These observations indicated heterogeneous participant-level response configurations that warranted descriptive multimodal examination.

To enhance analytical transparency, participant-level changes in ΔAIβ_2_ and ΔRMSSD across the auditory conditions are summarized in **Supplementary Table S1**. The descriptive response tendencies were assigned qualitatively to facilitate interpretation and should not be interpreted as formal classifications.

### 3.1 Group-level effects of auditory contexts

Across the three experimental contexts (silence, noise, and binaural stimulation), no consistent group-level main effects were observed for frontal β_2_ power, frontal asymmetry (AIβ_2_), or primary HRV indices (RMSSD, HF power). Non-parametric repeated-measures analyses did not reveal statistically significant condition differences (all p >.05).

Descriptive statistics nevertheless indicated substantial inter-individual variability in both cortical and autonomic responses across conditions. This variability was particularly evident in vagal indices (RMSSD and HF) and in frontal β_2_ modulation patterns. The absence of uniform group-level effects therefore reflected heterogeneous response directions rather than physiological stability.

### 3.2 Frontal β_2_ dynamics across auditory perturbations

Frontal upper-beta activity (21–30 Hz) demonstrated condition-dependent modulation patterns that varied across participants. At the descriptive level, both noise and binaural contexts were associated with moderate increases in right-frontal asymmetry relative to the silence baseline.

However, individual trajectories diverged markedly. In several participants, noise exposure produced increases in frontal β_2_ power accompanied by shifts toward right-frontal dominance. In contrast, a subset of participants exhibited β_2_ attenuation under noise, indicating reduced cortical arousal during the high-entropy auditory context.

Binaural-beat stimulation produced heterogeneous effects as well. In some individuals, frontal β_2_ activity showed partial normalization relative to noise exposure, whereas others displayed minimal cortical modulation across conditions.

### 3.3 Autonomic responses

Autonomic indices derived from HRV analysis revealed similarly heterogeneous patterns. Relative to silence, RMSSD decreased in 7 of 10 participants and increased in 3 of 10 participants. HF power showed a similar directional pattern (6/10 decreased, 4/10 increased), indicating reduced parasympathetic activity in several participants. However, in a smaller subset of individuals, noise exposure was accompanied by increased vagal indices.

Binaural stimulation produced increases in RMSSD and HF power in some participants, suggesting enhanced parasympathetic regulation during the structured auditory context. In other participants, autonomic indices remained relatively stable across conditions.

The variability observed in HRV responses paralleled the heterogeneity observed in frontal β_2_ dynamics.

### 3.4 Cortico–autonomic convergence patterns

To examine the relationship between cortical and autonomic responses, patterns of multimodal EEG-HRV convergence were evaluated at the individual level.

Across participants, heterogeneous multimodal EEG–HRV response configurations were observed, reflecting variability in the direction and magnitude of coordinated cortical and autonomic responses. In several individuals, increases in β_2_ power were accompanied by reductions in RMSSD, indicating cortico–autonomic divergence characterized by increased cortical arousal and reduced vagal tone. In contrast, other participants exhibited coordinated decreases in β_2_ activity together with increases in RMSSD, reflecting convergent regulation characterized by reduced cortical demand and increased parasympathetic engagement.

Descriptively similar directional tendencies were observed across epochs in some participants; however, temporal stability was not formally established.

The Results are presented in a stepwise manner, beginning with group-level analyses of EEG and HRV responses across the experimental conditions, followed by participant-level multimodal response characterization. Group-level statistical analyses are reported first to provide an overview of condition-dependent physiological changes, whereas subsequent sections focus on inter-individual variability and descriptive multimodal response patterns observed within participants. In keeping with the exploratory pilot design, the Results emphasize direct presentation of the observed data, while broader physiological interpretation is reserved for the Discussion.

**Figure 2:**
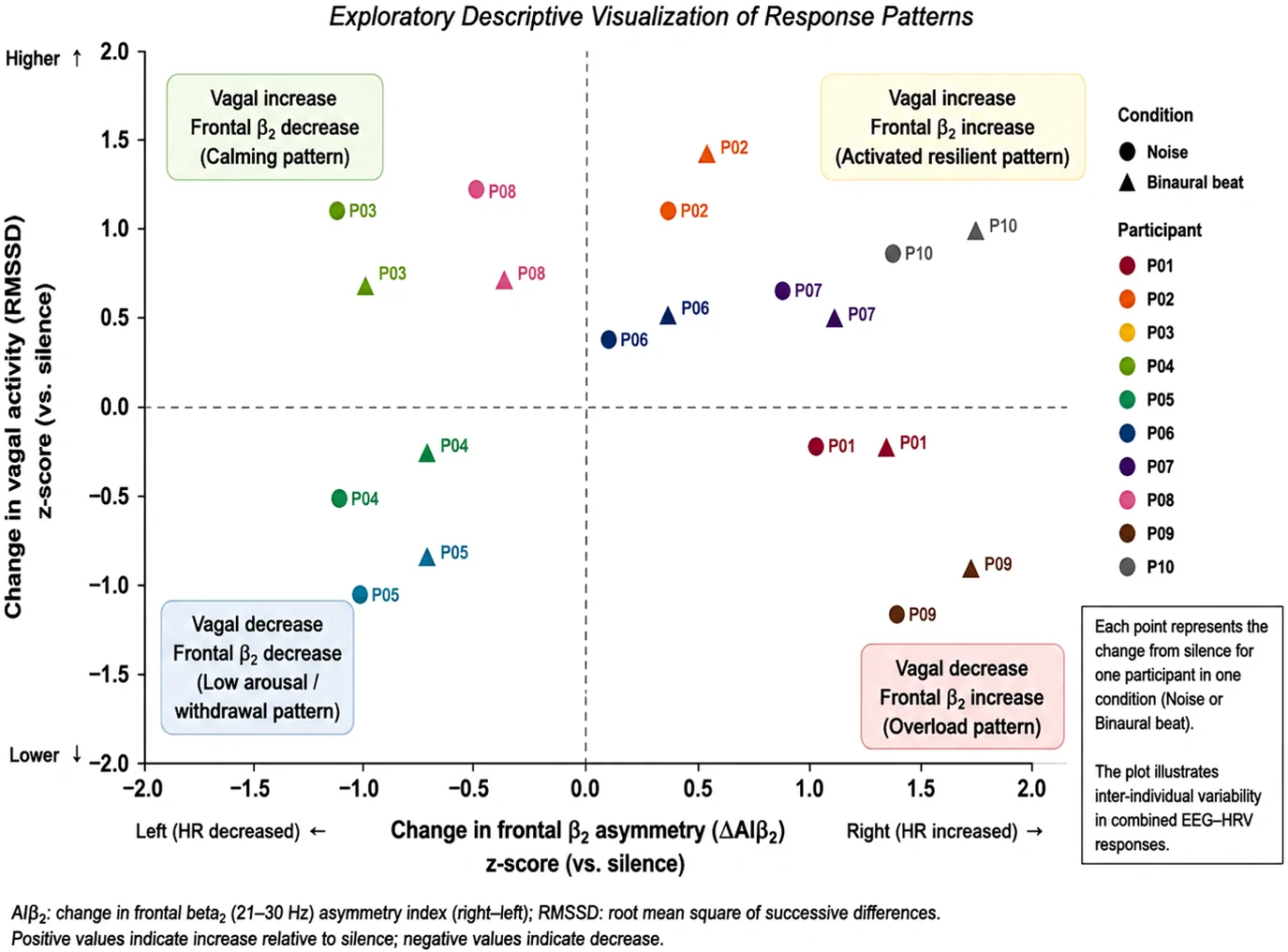
Participant-level cortico–autonomic response variability across auditory conditions. The scatter plot shows baseline-corrected changes in frontal β_2_ asymmetry (ΔAIβ_2_) and RMSSD (ΔRMSSD) for individual participants. Quadrant labels are provided solely as conceptual aids for describing response directionality and do not represent empirically derived subgroups, clustering results, or validated physiological phenotypes.

**Figure 2** illustrates the distribution of participant-level multimodal EEG–HRV responses observed under the standardized auditory conditions. The quadrants are intended as conceptual descriptors to facilitate physiological interpretation and should not be interpreted as empirically derived physiological subgroups or formal regulatory phenotypes.

Scatter plot illustrating the relationship between changes in frontal beta_2_ asymmetry (ΔAIβ_2_) and vagally mediated heart rate variability (ΔRMSSD) across individuals under different auditory conditions. Each data point represents an individual response profile, capturing coordinated shifts in cortical and autonomic activity relative to baseline.

The distribution of responses demonstrates substantial inter-individual variability in both the direction and magnitude of cortico–autonomic changes. Rather than forming a uniform cluster, data points are distributed across a multidimensional response space, indicating heterogeneous but structured coupling patterns.

Different regions of the response space can be descriptively interpreted as representing different multimodal response tendencies, including profiles characterized by increased cortical activation accompanied by reduced parasympathetic engagement, as well as profiles reflecting coordinated increases in vagal activity with reduced cortical load. Intermediate and stable response configurations are also observed, suggesting a continuum of regulatory modes rather than discrete categories.

Within a psychiatric framework, these patterns may reflect underlying differences in regulatory organization and contribute to the characterization of individual variability in brain–body dynamics. The observed coupling structure suggests that standardized perturbation paradigms may facilitate characterization of individual differences in coordinated brain–body responses. However, these findings should be regarded as exploratory and hypothesis-generating rather than evidence for empirically established physiological phenotypes.

To facilitate descriptive interpretation of the observed inter-individual variability, participant-level multimodal EEG–HRV response characteristics were summarized using conceptual response descriptors (**Table 1**). These descriptors were developed through qualitative evaluation of the combined EEG and HRV response profiles and are intended solely to organize the observed response tendencies within this exploratory pilot study. They do not represent empirically derived physiological subgroups, clustering results, or formal statistical classifications, but rather provide a descriptive framework for organizing and interpreting individual multimodal response variability.

**Table 1:** Conceptual characterization of multimodal EEG–HRV response patterns.

Cortical dynamics (β_2_)	Autonomic response (HRV)	Conceptual response descriptor	Descriptive physiological interpretation
β_2_ ↑	RMSSD ↓	High-reactive response pattern	Increased frontal β_2_ activity accompanied by reduced vagally mediated HRV, consistent with increased cortical demand and reduced parasympathetic engagement.
β_2_ ↓	RMSSD ↑	Stabilization-oriented response pattern	Reduced frontal β_2_ activity accompanied by increased vagally mediated HRV, consistent with reduced cortical demand and enhanced parasympathetic engagement.
β_2_ normalization	RMSSD ↑	Recovery-associated response pattern	Partial normalization of frontal β_2_ activity accompanied by increased vagally mediated HRV, consistent with recovery-oriented physiological regulation.
Minimal β_2_ change	Minimal HRV change	Minimal-response pattern	Limited multimodal physiological change across the experimental conditions, suggesting relatively stable physiological regulation under the present experimental paradigm.

The response descriptors summarize direction-specific participant-level EEG–HRV tendencies observed under the standardized auditory conditions. These conceptual descriptors are intended solely to facilitate descriptive interpretation of multimodal response variability. They were not derived through clustering or formal classification procedures and should not be interpreted as empirically validated physiological phenotypes or discrete biological subgroups.

To further improve analytical transparency, participant-level changes in ΔAIβ_2_ and ΔRMSSD across both auditory conditions are provided in **Supplementary Table 1**. The descriptive response tendencies summarize the observed multimodal response configurations and are intended solely to facilitate interpretation of individual physiological variability. They should not be interpreted as formal physiological classifications or empirically derived phenotypes.

## 4 Discussion

The present findings should be interpreted within the broader context of ongoing efforts to establish objective, physiology-based markers in psychiatry. Prior research has demonstrated that EEG-derived measures can capture clinically relevant variability and support biologically informed stratification (9, 10, 26). From a clinical perspective, increasing attention has been directed toward identifying biologically meaningful dimensions underlying psychiatric variability, moving beyond categorical diagnoses toward dimensional and systems-level characterization.

However, most existing approaches remain predominantly brain-centric and rely on static or resting-state measurements. While theoretical models have emphasized the importance of brain–heart interactions (11), empirical implementations integrating central and autonomic dynamics in a unified, response-based framework remain limited. The present findings are consistent with the possibility that combined EEG and HRV measures acquired during standardized auditory perturbations may provide complementary information regarding individual cortico–autonomic responses. Rather than reflecting static traits, these patterns may capture dynamic regulatory tendencies that emerge in response to external modulation (14).

Such an approach may contribute to the development of physiology-informed, personalized assessment strategies, particularly in the context of psychiatric heterogeneity where individual differences in regulatory processes are increasingly recognized as a key factor (1, 27, 30).

The present study investigated whether brief standardized auditory perturbations can characterize individual cortico–autonomic response variability using synchronized EEG and HRV measurements. Rather than producing uniform group-level physiological responses, the results revealed heterogeneous within-person configurations of cortical and autonomic dynamics. These observations are consistent with the possibility that individuals differ in multimodal physiological response tendencies under standardized perturbation conditions.

Consistent with neurovisceral integration theory, the observed coupling patterns between frontal β_2_ dynamics and vagally mediated HRV markers suggest coordinated interactions between cortical arousal systems and parasympathetic regulation (5, 11). Within this framework, prefrontal cortical activity is thought to modulate autonomic flexibility through descending regulatory pathways, enabling adaptive adjustments of physiological state in response to environmental demands. The present findings are consistent with this theoretical framework and illustrate how synchronized EEG–HRV measurements may capture coordinated brain–body responses during standardized perturbations.

Importantly, the absence of significant group-level effects across auditory contexts does not indicate a lack of physiological response. Rather, the results reveal that identical sensory inputs can produce divergent regulatory trajectories across individuals. This observation aligns with growing evidence that emotional and attentional responses to auditory stimulation vary substantially across listeners (15, 24). Instead of reflecting simple stimulus valence or subjective preference, the patterns observed here suggest that auditory contexts may act as probes amplifying pre-existing regulatory tendencies within the cortico–autonomic system.

The observed multimodal response patterns between frontal β_2_ modulation and vagal change further is consistent with heterogeneous cortico–autonomic response tendencies across participants.

In several participants, increases in cortical arousal were accompanied by vagal withdrawal, consistent with high-engagement regulatory responses characterized by enhanced attentional activation and reduced parasympathetic tone. In contrast, other individuals exhibited coordinated reductions in cortical demand together with vagal recruitment, reflecting stabilization-oriented regulatory responses. The descriptive similarity of some responses across epochs raises the possibility of within-session consistency, although the present design does not establish temporal stability or trait-like regulatory characteristics.

These observations are consistent with theoretical perspectives emphasizing network-level interactions between cortical control systems and autonomic regulation in shaping adaptive behavior (17, 18). Frontal beta-band dynamics have been associated with attentional gating and arousal scaling (20), while HRV indices provide established markers of parasympathetic flexibility and emotional regulation capacity (6, 7). By integrating these modalities within a single wearable framework, the present study demonstrates that coordinated cortical and autonomic dynamics can reveal structured regulatory architectures at the individual level.

From a methodological perspective, the findings highlight the potential of wearable multimodal neurophysiology for investigating individualized regulatory organization. Portable EEG and HRV systems enable simultaneous assessment of brain–body dynamics under ecologically relevant conditions (22, 23). When combined with structured sensory perturbations, such approaches provide scalable tools for characterizing individual multimodal response variability and examining individual differences in affective regulation.

The present study provides an initial demonstration of multimodal cortico–autonomic regulatory patterns using a controlled perturbation framework. The within-subject design enables detailed characterization of individual-level dynamics, while the use of a wearable EEG system supports ecologically relevant, low-burden, and scalable data acquisition. This approach allows repeated and context-sensitive assessment of regulatory responses across conditions. Wearable EEG systems may therefore offer a scalable and ecologically valid complement to conventional laboratory-based neurophysiological assessments, facilitating the investigation of individualized cortico–autonomic regulation across diverse contexts.

Within this framework, affective responsiveness may be understood as emerging from intrinsic cortico–autonomic regulatory architectures rather than solely from transient stimulus-driven modulation. Coordinated multimodal response patterns may therefore represent fundamental units of regulatory organization linking cortical arousal dynamics and parasympathetic flexibility.

Taken together, the present findings suggest that affective responsiveness may be structured by intrinsic cortico–autonomic regulatory architectures rather than solely by transient stimulus-driven state modulation (5). These findings are consistent with the possibility that individuals differ in cortico–autonomic regulatory parameters governing the relationship between cortical arousal signals and autonomic adjustments.

Detailed individual-level response trajectories are reported in a complementary analysis focusing on participant-specific physiological profiles. Together, these findings suggest that brief sensory perturbations may provide a scalable experimental approach for revealing individual regulatory response tendencies, offering a promising framework for investigating individual differences in affective regulation and brain–body integration within contemporary affective neuroscience.

Overall, the present findings are consistent with the exploratory hypotheses and support further investigation of perturbation-based multimodal EEG–HRV assessment as a framework for characterizing individual cortico–autonomic response variability in future studies.

### Strengths and Limitations

The present study has several methodological strengths. First, it employed a synchronized multimodal EEG–HRV approach that enabled simultaneous assessment of cortical and autonomic responses during standardized auditory perturbations. Second, the within-subject experimental design reduced inter-individual methodological variability and allowed detailed characterization of participant-level physiological responses across identical experimental conditions. Third, the use of wearable neurophysiological technology supports the feasibility of scalable and ecologically applicable multimodal assessments, providing a practical framework for future investigations of brain–body regulation.

Several limitations should also be acknowledged. The study was designed as an exploratory pilot investigation and included a small sample of healthy participants with a relatively wide age range. Consequently, the findings should be interpreted cautiously and should not be considered evidence for empirically validated physiological subgroups or clinically applicable classifications. Rather than performing clustering analyses or formal phenotype identification, the present study used conceptual response descriptors to organize the observed multimodal EEG–HRV response variability. Furthermore, the observed response patterns require replication in larger and clinically diverse populations using quantitative subgroup identification methods, longitudinal designs, and independent validation cohorts before conclusions regarding stable physiological profiles or precision psychiatry applications can be drawn.

Accordingly, the principal contribution of the present study is methodological rather than classificatory. The findings demonstrate the feasibility of a perturbation-based multimodal EEG–HRV framework for characterizing individual cortico–autonomic response variability and provide a hypothesis-generating foundation for future confirmatory research in physiology-informed precision psychiatry.

Future studies incorporating larger cohorts, data-driven clustering approaches, and multimodal machine-learning models will be necessary to determine whether the candidate response patterns observed here correspond to reproducible physiological subgroups.

A complementary analysis of participant-level frontal β_2_ asymmetry trajectories from the same experimental paradigm is reported separately, whereas the present study focuses on the integrated characterization of multimodal EEG–HRV response variability.

## 5 Conclusion

The present findings suggest that brief standardized auditory perturbations can characterize individual cortico–autonomic response variability beyond transient state-related physiological changes.

The observed multimodal response patterns indicate substantial inter-individual variability in coordinated cortical and autonomic responses under standardized auditory conditions.

Within this perspective, perturbation-based multimodal EEG–HRV assessment offers a scalable methodological framework for characterizing coordinated brain–body responses and may support future physiology-informed approaches to individualized assessment in precision psychiatry.

Future studies involving larger and clinically diverse cohorts, together with data-driven subgroup identification methods, will be required to determine whether the candidate response patterns observed in this exploratory pilot study correspond to reproducible physiological subgroups.

## Data Availability

The raw data supporting the conclusions of this article will be made available by the authors, without undue reservation.
