# Supplementary material for: Multimodal EEG–HRV assessment of individual regulatory patterns: a perturbation-based approach toward precision psychiatry
Source: Front Psychiatry. 2026 Aug 24;17:1851256. doi: 10.3389/fpsyt.2026.1851256 (PMC13547485; doi:10.3389/fpsyt.2026.1851256)
Supplement: Supplementary file 1 [file DataSheet1.pdf]

Participant ID	$\Delta AI\beta_2$ (Noise)	ΔRMSSD (Noise), ms	$\Delta AI\beta_2$ (Binaural)	ΔRMSSD (Binaural), ms	Conceptual multimodal response descriptor
**P01**	+0.104	+1.0	+0.344	−1.0	Predominantly cortical modulation with minimal autonomic change
**P02**	+0.064	+0.6	−0.014	−1.9	Minimal multimodal modulation
**P03**	+0.264	−25.3	+0.771	−22.2	Rightward cortical modulation accompanied by vagal withdrawal
**P04**	+0.025	−4.4	−0.015	−8.4	Predominantly autonomic modulation with minimal cortical change
**P05**	−0.591	−8.3	−0.278	−7.8	Leftward cortical modulation with reduced vagal activity
**P06**	+0.741	+8.9	+0.770	−7.7	Sustained cortical modulation with condition-dependent autonomic regulation
**P07**	+0.575	+7.2	+0.576	−11.0	Sustained cortical modulation with condition-dependent autonomic regulation
**P08**	−0.438	−10.5	−0.607	−10.0	Leftward cortical modulation with vagal withdrawal
**P09**	−0.279	+14.7	+0.256	−0.1	Mixed multimodal response across auditory conditions
**P10**	+0.629	+1.1	+0.553	+30.7	Binaural-associated vagal recruitment with sustained cortical modulation

Supplementary Table S1. Participant-level baseline-corrected changes in frontal β_2 asymmetry ($\Delta AI\beta_2$) and vagally mediated heart rate variability (ΔRMSSD) during noise-music and binaural-beat auditory stimulation relative to the silence condition.

$\Delta AI\beta_2$ values were calculated using the predefined log-transformed frontal β_2 asymmetry index [$AI\beta_2 = \ln(AF8\beta_2) - \ln(AF7\beta_2)$] and expressed as changes relative to the silence condition. ΔRMSSD values represent within-participant changes relative to the corresponding silence baseline.

The conceptual multimodal response descriptors summarize the observed multimodal EEG–HRV response configurations and are intended solely to facilitate interpretation of participant-level physiological variability. They were assigned qualitatively and were not derived using clustering algorithms, machine-learning approaches, or formal statistical classification

procedures. Accordingly, they should not be interpreted as empirically validated physiological subgroups, biomarkers, diagnostic categories, or regulatory phenotypes.

The conceptual response descriptors were assigned following qualitative integration of participant-level EEG and HRV response patterns across the experimental conditions.

Positive $\Delta AI\beta_2$ values indicate a relative right-frontal shift, whereas negative values indicate a relative left-frontal shift. Positive ΔRMSSD values indicate increased vagally mediated heart rate variability relative to the silence condition, whereas negative values indicate reduced vagally mediated heart rate variability.
